# Clinical and Sleep Characteristics and the Effect of CPAP Treatment on Obese Patients with Obstructive Sleep Apnea and Asthma—A Retrospective Study

**DOI:** 10.3390/healthcare13172240

**Published:** 2025-09-08

**Authors:** Konstantina Chadia, Kostas Archontogeorgis, Fotios Drakopanagiotakis, Konstantinos Bonelis, Stavros Anevlavis, Paschalis Steiropoulos

**Affiliations:** 1 MSc Program in Sleep Medicine, Medical School, Democritus University of Thrace, 68100 Alexandroupolis, Greece; con_chadia@yahoo.gr (K.C.); k.archontogeorgis@yahoo.it (K.A.); 2 Department of Pneumonology, Medical School, Democritus University of Thrace, 68100 Alexandroupolis, Greece; fdrakopanagiotakis@gmail.com (F.D.); kon.bonelis@gmail.com (K.B.); anevlavis@yahoo.com (S.A.)

**Keywords:** obstructive sleep apnea, asthma, continuous positive airway treatment, asthma control, comorbidities

## Abstract

Introduction: Patients with obstructive sleep apnea (OSA) and asthma share common symptoms and risk factors. Aim: The aim of this study is to evaluate the clinical characteristics of patients with OSA and asthma and assess the impact of CPAP treatment on asthma control and exacerbations. Methods: Consecutive patients diagnosed with concomitant OSA and asthma were enrolled in the study. Data on patients’ characteristics, respiratory function during wakefulness, and polysomnography were recorded. Additionally, asthma control and exacerbation history were assessed the year before and after initiation of CPAP therapy. Results: The cohort included 102 patients (53 men and 49 women; mean age 56.5 ± 12.8 years). The severity of OSA was classified as severe in 49%, moderate in 27.5%, and mild in 23.5% of patients. The most common comorbidities were arterial hypertension (66.7%) and dyslipidemia (52%). Before CPAP initiation, most patients (55.9%) had moderate asthma control (ACT score 17.4 ± 0.9). Following CPAP treatment, the ACT score was improved (*p* < 0.001) and asthma exacerbations were significantly reduced (*p* = 0.002). Moreover, the Asthma Control Test (ACT) score was negatively correlated with BMI (r = −0.209, *p* = 0.035), AHI (r = −0.426, *p* < 0.001), oxygen desaturation index (r = −0.466, *p* < 0.001), and percentage of sleep time with oxygen saturation <90% (T < 90%) (r = −0.228, *p* = 0.021). Also, patients who experienced exacerbations (44/102) had higher AHI (*p* = 0.022) and more severe nocturnal hypoxia (T < 90%, *p* = 0.016). Conclusions: Asthma control is associated with OSA severity and BMI, while CPAP therapy seems to improve asthma control and reduces exacerbations in patients with concomitant OSA and asthma.

## 1. Introduction

Obstructive sleep apnea (OSA) is the most common and well-described form of sleep-disordered breathing, affecting approximately one billion people aged 30 to 69 years worldwide [1,2]. It is characterized by repetitive episodes of complete or partial upper airway collapse, leading to oxygen desaturation during sleep, frequent arousals, and sleep fragmentation [3], with patients typically experiencing both daytime and nighttime symptoms, such as excessive daytime sleepiness (EDS), loud persistent snoring, and breathing pauses [4]. Polysomnography (PSG) is the gold standard for OSA diagnosis and continuous positive airway pressure (CPAP) is the treatment of choice for moderate-to-severe cases [5]. Although oral appliances, like mandibular advancement devices, and surgical options, such as maxillomandibular advancement, are the preferred choices for patients with mild-to-moderate OSA, in some cases they are suitable for patients who cannot tolerate CPAP or for those with anatomical abnormalities; and as one size does not fit all, a combination therapy is still an option, as well as lifestyle interventions [6,7].

Asthma, on the other hand, is a long-term respiratory disease that is currently turning into a public health issue, affecting approximately 5% (~300 million) patients globally [8] and contributing to nearly 420,000 deaths annually [9]. According to the Global Initiative for Asthma (GINA), asthma is characterized by chronic airway inflammation leading to airway narrowing and air flow limitation and causing recurrent respiratory symptoms, such as wheezing, shortness of breath, chest tightness, and coughing [10]. Symptoms often worsen at night and can be related to severe exacerbations. Asthma is believed to be the result of complex interactions between genetic and environmental factors and it is closely related to bronchial hyperresponsiveness [11].

Furthermore, asthma has been identified as an independent risk factor for OSA [12]. More specifically, its duration is a recognizable predisposing factor for newly diagnosed OSA and EDS [13]. In addition, asthma control and an increased risk of asthma exacerbations has been linked to OSA [14]. Studies indicate that approximately 49% of patients with moderate-to-severe and poorly controlled asthma also have moderate-to-severe OSA [15]. According to the European Sleep Apnea Database (ESADA), the prevalence of OSA is estimated to be approximately 95% among patients with severe asthma, yet only 5% of OSA patients are officially diagnosed with concomitant asthma [16,17].

As studies indicate that asthma and OSA share overlapping symptoms and risk factors, like rhinitis, gastroesophageal reflux disease (GERD) and obesity [18], recent research focused on these interactions, which are commonly referred to as alternative overlap syndrome (AOS) [19]. Although a multitude of studies have explored the relationship between asthma and OSA, the precise effect of CPAP treatment in these patients remains unclear, as the available data are inconsistent, and no definitive link has been firmly established. A possible explanation for these discrepancies may be attributed to differences in patient populations, particularly the severity of OSA, as well as the uncertain impact of CPAP on bronchial hyperresponsiveness.

The aim of the current study was to investigate both the clinical characteristics of patients with AOS and evaluate the effects of CPAP treatment on asthma control and exacerbations in patients with AOS.

## 2. Materials and Methods

### 2.1. Patients

Consecutive patients with physician-diagnosed asthma who were referred to the sleep laboratory of our institution between November 2014 to April 2024 and diagnosed with OSA were enrolled in the study. All participants had signed written informed consent. The study protocol for the retrospective use of data was approved by the hospital’s institutional review board (approval date: 11 July 2024) and it was in accordance with the Helsinki Declaration of Human Rights [20]. The exclusion criteria were (a) age under 18 years old, (b) refusal to provide a written informed consent, and (c) patients with central sleep apneas.

Patients’ anthropometric characteristics [height; weight; neck, hip, and waist circumference; waist/hip circumference ratio; body mass index (BMI)] comorbidities, and current asthma therapy were recorded. Respiratory function testing was performed via the Master Screen Body apparatus (Erich Jaeger GmbH, Wuerzburg, Germany) and patients were assessed regarding asthma control and history of exacerbations. All participants underwent an in-laboratory overnight polysomnography and a Greek version of the Epworth sleepiness scale (ESS) was used to determine excessive daytime sleepiness. ESS scores >10 were considered indicative of EDS [21].

The validated Greek version of the Asthma Control Test (ACT) [22] was used to assess patients’ asthma control over the last 4 weeks, the year before and after initiation of CPAP therapy. The ACT consists of 5 questions and scores under 15 indicate uncontrolled, scores from 15 to 19 indicate partially controlled, and scores over 20 indicate well-controlled asthma. Asthma exacerbation was defined according to the GINA 2024 guidelines as a progression of main asthma symptoms and a decline in lung function from the patient’s usual status, especially a decrease in Peak Expiratory Flow (PEF) over 60% of the patient’s personal best or predicted score [10].

OSA diagnosis was established by polysomnographic results, either as apnea-hypopnea index (AHI) ≥ 5 events/hour of sleep accompanied with symptoms or as AHI ≥ 15 events/hour of sleep [4]. Adherence to CPAP therapy was defined based on the American Thoracic Society Statement as use of the device for at least 4 h per night for 70% or more of the nights within a given period, typically 30 consecutive days during the first trimester [23].

### 2.2. Polysomnography

An overnight PSG study was performed from 22:00 to 06:00 (Alice^®^ 4, Philips Respironics, Murrysville, PA, USA). Channels for brain activity (electroencephalogram), eye movements (electro-oculogram), muscle activity or skeletal muscle activation (electromyogram) and heart rhythm (electrocardiogram) were used for sleep stage determination. Respiratory airflow (nasal/oral flow) and respiratory effort indicators were added, along with peripheral pulse oximetry.

Manual scoring of respiratory events and sleep stages was performed in accordance with American Academy of Sleep Medicine guidelines (AASM) [24]. Specifically, a ≥90% of reduction in airflow for at least 10 s was defined as apnea and a ≥30% reduction in airflow for at least 10 s accompanied by oxygen desaturation of at least 3% or an arousal registered by the electroencephalogram was defined as hypopnea [24]. The AHI was calculated based on the average number of apneas and hypopneas per hour of total sleep recorded [24]. The average number of oxyhemoglobin desaturation episodes per hour of total sleep was defined as the oxygen desaturation index (ODI) [24].

### 2.3. Statistical Analysis

Statistical analysis was carried out using the IBM Statistical Package for Social Sciences (SPSS Inc. Released 2008. SPSS Statistics for Windows, Version 17.0. Chicago, IL, USA: SPSS Inc.). The Shapiro–Wilk test was used for the normality of distribution for continuous variables. All data are expressed as the mean ± standard deviation. Comparison of percentages between groups was performed with the chi-square test. Correlations were analyzed with Pearson’s correlation coefficient in normally distributed variables. Comparisons between groups were performed using Student’s *t*-test and one-way analysis of variance (ANOVA); post hoc analysis was performed using Tukey’s test. Statistical significance was defined at *p* < 0.05.

## 3. Results

Overall, 102 patients with AOS (53 males and 49 females) with a median age of 56.6 ± 12.8 years and moderate-to-severe obesity (BMI 38.2 ± 8 kg/m^2^) were enrolled in the study. In total, 45 patients were nonsmokers, 29 were former smokers, and 28 were still smoking. The majority of women included (45/49) were postmenopausal. All anthropometric characteristics are shown in Table 1.

Arterial hypertension (66.7%), dyslipidemia (52%), diabetes mellitus (46.1%), allergic rhinitis (45.1%), hypothyroidism (30.4%), and gastroesophageal reflux (29.4%) were the most frequently reported comorbidities).

With regard to sleep parameters, severe, moderate, and mild OSA was observed in 49%, 27.5%, and 23.5% of patients, respectively. The majority of the patients (90.2%) were receiving a fixed dose combination of inhaled corticosteroid plus a long-acting beta_2_-agonist. Good adherence to CPAP therapy was recorded for 73/102 patients (71.6%). Before the initiation of CPAP treatment, most participants (58/102) had partially controlled asthma (ACT score 17.4 ± 0.9), while those with uncontrolled asthma had a higher AHI (*p* < 0.001) and oxygen desaturation index (*p* < 0.001) and they reported higher levels of daytime sleepiness (an ESS score of 12.4 ± 5 for patients with uncontrolled asthma vs. 8.6 ± 4.9 for patients with partially controlled asthma, and *p* = 0.003; 12.4 ± 5 for patients with uncontrolled asthma vs. 7.5 ± 4.5 for patients with well controlled asthma, *p* = 0.004). A comparison of the sleep characteristics of patients with uncontrolled, partially controlled, and well-controlled asthma is presented in Table 2.

**Figure 1:**
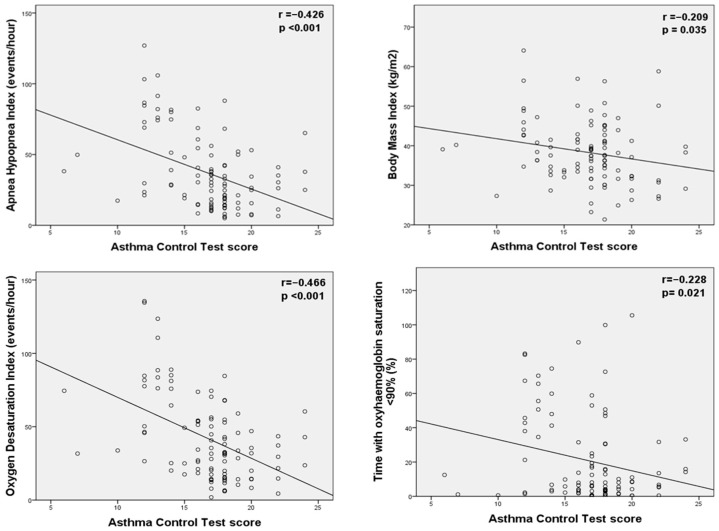
Negative correlations between ACT score and AHI, BMI, oxygen desaturation index, and T < 90%. Abbreviations: ACT: asthma control test; FEV_1_: forced expiratory volume in the first second; FVC: forced vital capacity; PEF: peak expiratory flow.

Although no statistically significant difference was found in anthropometric characteristics and respiratory function between patients with uncontrolled, partially controlled, and well-controlled asthma (Table 3), further analysis showed that ACT score was negatively correlated with BMI (r = −0.209, *p* = 0.035), AHI (r = −0.426, *p* < 0.001), ODI (r = −0.466, *p* < 0.001), and time spent with oxyhemoglobin saturation < 90% (r = −0.228, *p* = 0.021) (Figure 1). After CPAP treatment, fewer asthma exacerbations were observed (*p* = 0.002), and the ACT score was improved (16.8 ± 3.2 before vs. 20.5 ± 2.6 after CPAP, *p* < 0.001), especially in patients with good compliance (≥4 h/night) with CPAP (19.6 ± 3 for patients with non-adherence to CPAP vs. 20.8 ± 2.4 for patients with adherence to CPAP use, *p* = 0.038).

In addition, patients with exacerbations (44/102) suffered more frequently from allergic rhinitis (*p* = 0.001) and had higher AHI (43.7 ± 28.7 vs. 31.6 ± 23.7, *p* = 0.022) and more severe nocturnal hypoxia (T < 90%, 28.3 ± 31.1 vs. 15 ± 19.4% of recording time, *p* = 0.016). A comparison of the sleep characteristics of patients with and without asthma exacerbations is presented in Table 4.

## 4. Discussion

The present study aimed to define the clinical characteristics and the impact of CPAP treatment on asthma control and exacerbations in patients with AOS. The patients included in the study were middle-aged and obese, with comorbidities associated with metabolic syndrome. Patients with uncontrolled asthma and exacerbations had more severe OSA and the respiratory indices during sleep were worse, while a negative correlation between ACT score and BMI was observed. Finally, CPAP treatment and good compliance led to significant improvements in asthma control and fewer asthma exacerbations.

Numerous studies have examined the interactions between OSA and asthma, primarily focusing on how OSA exacerbates asthma symptoms [25]. However, some studies identify asthma as a clinical syndrome that contributes to both the onset and worsening of OSA through shared pathophysiological mechanisms, risk factors, and upper-airway anatomy [26].

Obesity is a well-recognized aggravating factor for both asthma and OSA. Asthmatic patients tend to have higher obesity rates due to reduced physical activity and the frequent use of oral corticosteroids [27], and sleep apnea is associated with an increased risk of central obesity, cardiovascular disease, resistant hypertension, and type 2 diabetes [28]. In a meta-analysis, Kong et al. [29] found that patients with both OSA and asthma had a higher BMI compared to those with asthma alone (mean difference: 2.15 [3.64, 0.67] kg/m^2^; *p*  =  0.004). Similarly, a hospital-based study by Lin et al. [30], reported that 93 out of 238 patients with OSA and asthma had a higher BMI (*p* < 0.001) compared to patients without concomitant OSA, while Shen et al. [31] found that the coexistence of asthma and obesity significantly increased the occurrence of OSA in asthma patients (adjusted HR: 6.07, 95% CI = 3.24–11.4, *p* < 0.001). Additionally, Huang et al. [32] observed that both BMI and waist circumference were independently and strongly associated with OSA.

Contradicting most epidemiological studies, Al-Lawati et al. [33] found no significant difference in mean BMI between patients with uncontrolled and well-controlled asthma. Moreover, in the study by Laforest et al. [34], BMI was identified as an independent factor associated with inadequate asthma control, consistent with the findings of Schatz et al. [35] who evaluated asthma control in 570 adult patients using the ACT questionnaire and demonstrated an independent negative correlation between BMI and ACT score (*p* = 0.01). Similarly, data from the European National Health and Wellness Survey indicated that patients with well-controlled asthma had a lower prevalence of BMI ≥ 30 kg/m^2^ compared to those with poorly controlled asthma (22.7% vs. 30.0, *p* < 0.001) [36]. These findings are in line with our study, which also demonstrated a negative correlation between ACT score and BMI, emphasizing the critical role of obesity in asthma control, particularly in patients with AOS.

In our study, arterial hypertension, dyslipidemia, and diabetes mellitus were the most prevalent comorbidities. A population-based study of 74,342 Canadian adults reported that patients with asthma had a higher risk of cardiovascular disease compared to non-asthmatic individuals (43% for heart disease and 36% for hypertension) [37]. Consequently, patients with AOS tend to exhibit a higher Charlson comorbidity index than those with OSA alone (2.3 ± 0.2 vs. 1.9 ± 1.8, *p* < 0.0001 [38]. These findings align with existing literature, reinforcing that patients with AOS are more susceptible to cardiometabolic complications, and therefore face an increased risk of morbidity and mortality.

In addition to metabolic syndrome, allergic rhinitis was another commonly reported comorbidity in our study population, with a higher prevalence among patients experiencing asthma exacerbations. These findings suggests that upper-airway diseases may independently contribute to the risk of asthma attacks [14]. Additionally, asthma comorbidities are believed to affect not only the severity and characteristics of sleep apnea, but also the degree of daytime sleepiness. To emphasize the overlap of shared comorbidities and underlying pathophysiological mechanisms between rhinitis and OSA, Kasasbeh et al. [39] proposed the term ‘CORE Syndrome’ (Cough, Obesity/OSA, Rhinosinusitis, and Esophageal reflux). Notably, nasal sinus diseases, including allergic rhinitis, have been identified as independent risk factors for both asthma exacerbations and excessive daytime sleepiness [39], particularly in patients with difficult-to-treat asthma [14].

Although the direct correlation between the degree of nasal obstruction and the severity of sleep apnea remains unclear, rhinitis has been recognized as a risk factor for elevated AHI in the general population [40]. While Kramer et al. [41] found no significant differences in sleep characteristics between patients with and without allergic rhinitis, Braido et al. [42] reported, in a cross-sectional study of 740 asthma-only patients and 1201 patients with both asthma and allergic rhinitis, that the presence of allergic rhinitis increased the risk of OSA by 1.44-fold. Similar results were described by Lin et al., who demonstrated a significantly higher prevalence of allergic rhinitis (*p* < 0.001) in a group of 93 patients with AOS [30]. With regard to sleep parameters, Kalpaklıoglu et al. [43] showed that both patients with and without allergic rhinitis experienced frequent arousals during sleep. However, more severe sleep apnea was noted in those without allergic rhinitis [43]. Furthermore, this group was at an increased risk of developing OSA regardless of asthma coexistence (odds ratio 6.4).

Nearly half of the participants in our study had severe OSA and partially controlled asthma. The meta-analysis by Wang et al. [15] demonstrated that asthma severity increases the risk of OSA (pooled OR  =  4.36, 95% CI 2.49–7.64, I^2^  =  74.2%, *p*  < 0.001) but does not significantly impact OSA severity, based on sleep parameters (weighted mean difference (WMD) = 0.60, 95% CI 0.16–1.04), except for excessive daytime sleepiness (pooled WMD  =  0.60, 95%CI 0.16–1.04, *p*  =  0.007). One possible explanation for this finding is the lower arousal threshold observed in patients with AOS compared to those with OSA alone [44]. In addition, respiratory muscle weakness, instability of the respiratory drive, and nocturnal asthma symptoms lead to frequent arousals, sleep fragmentation, and reduced total sleep [45]. These pathophysiological mechanisms may reduce the arousal threshold in sleep apnea and result in lower AHI [46]. However, the polysomnography results in the studies included in the meta-analysis of Wang et al. [15] may have been influenced by different sleep disturbances due to asthma severity, which was not consistently recorded, representing a key limitation of the study.

In a recent cross-sectional study, Sundbom et al. [47] examined the correlation between asthma, OSA, and nocturnal oxygen saturation. Their findings showed that asthma patients exhibited nocturnal hypoxemia compared to non-asthmatic patients (93.8% vs. 94.3%, *p* = 0.01). However, this difference could not be solely attributed to concomitant OSA, suggesting a multifactorial etiology of oxygen desaturation during sleep in these patients. Furthermore, a community-based study in a female population [48] revealed that the coexistence of asthma and sleep apnea is associated with poorer sleep quality and more severe nocturnal hypoxemia, independent of each other. Specifically, patients with AOS had a lower average oxyhemoglobin saturation compared to asthma patients without sleep apnea (93.4% vs. 94.7%, *p* = 0.04). In addition, Lin et al. [30] conducted a study including 238 participants, divided into two groups: an observation group (93 patients with both asthma and sleep apnea) and a control group. They found that within the observation group, patients with severe OSA had a higher AHI and spent more time with oxyhemoglobin saturation <90% compared to those with mild and moderate sleep apnea (*p* < 0.05).

Although OSA is associated with low oxyhemoglobin saturation, the impact of hypoxemia on asthma control has yet to be determined. Sariman et al. [49] demonstrated that air wall thickening, a key factor in asthmatic patients linked to bronchial hyperresponsiveness, is positively correlated to AHI, nocturnal hypoxemia, and the percentage of time spent with oxyhemoglobin saturation <90%. In another study by Jordan et al. [50], a longer period of diagnosed OSA was recorded in participants with worse asthma control (40.6%, 34.2%, and 17.6% in patients with uncontrolled, partially controlled and well-controlled asthma, respectively, *p* < 0.001). These findings align with those of Zidan et al. [51], who also reported a significant association between OSA and uncontrolled asthma. Our study supports these results, as most patients had partially controlled asthma, while those with uncontrolled asthma exhibited a higher AHI and oxygen desaturation index. Moreover, we identified a negative association between asthma control levels and both the oxygen desaturation index and the percentage of time spent with oxyhemoglobin saturation <90%.

Additionally, while mild hypercapnia is well tolerated by asthmatic patients, severe hypercapnia can disrupt alveolar ventilation, potentially leading to significant implications for patients that may necessitate mechanical ventilation in cases of severe exacerbations [52]. This disruption in alveolar ventilation among asthmatics may also explain why individuals with frequent exacerbations present with more severe OSA, with a higher AHI, and significant hypoxemia during sleep compared to those without exacerbations. Therefore, the destabilization of the respiratory system, the responses to hypercapnic stimuli, and the loop gain theory may help explain the results observed in our study in patients with AOS and asthma exacerbations. These findings are consistent with the available literature. Wang et al. [53] reported an increase in severe asthma exacerbations in patients with a higher AHI (odds ratio 1.322, 95% CI 1.148–1.523, *p* < 0.001). Moreover, in an observational study, Yii et al. [54] identified OSA as a risk factor for recurrent asthma attacks.

The impact of CPAP therapy, the gold standard treatment for moderate and severe OSA, on asthma control is controversial. Results from a questionnaire-based study including 152 female patients in whom asthma treatment was initiated before CPAP [55] revealed a statistically significant improvement in asthma symptoms and ACT score, along with a reduction in rescue medication use (*p* < 0.001). In a more recent study, 100 patients with asthma were referred for sleep assessment [56]. The majority (54%) were diagnosed with severe OSA and had either partially controlled (29%) or uncontrolled (30%) asthma. After at least 3 months of CPAP therapy, nearly all patients (90%) had good adherence to treatment, and over half reported an improvement in asthma symptoms, as witnessed by a statistically significant increase in ACT score (ACT score 19 ± 4 before CPAP use and 21 ± 4 after 3 or more months of CPAP use, *p* < 0.001). Similarly, a prospective study by Serrano-Pariente et al. showed that quality of life and asthma control were both improved after 6 months of CPAP therapy [57]. Specifically, the ACQ score decreased from 1.39 ± 0.91 before CPAP initiation to 1.0 ± 0.78 after 6 months of CPAP treatment, while the proportion of patients with uncontrolled asthma decreased from 41.4% at baseline to 17.2% at the study’s conclusion (*p* = 0.006). Furthermore, Cifti et al. [58] reported a significant improvement in nocturnal asthma symptoms after two months of CPAP use in 16 patients with AOS and persistent nocturnal symptoms (*p* < 0.05). Regarding asthma exacerbations, Serrano-Pariente et al. [57] recorded a decrease in the number of patients experiencing asthma exacerbations from 35.4% to 17.2% after 6 months of CPAP therapy (*p* = 0.015). This effect was particularly pronounced in patients with moderate-to-severe OSA and those adherent to CPAP therapy. These findings are consistent with our study’s results, highlighting CPAP therapy as a potentially modifiable factor in improving clinical symptoms and preventing asthma exacerbations in patients with severe asthma. Undoubtedly, the findings of our study should be interpreted in light of certain limitations. Firstly, we did not account for whether patients were receiving additional treatments for their comorbidities, such as allergic rhinitis, alongside CPAP therapy. These treatments could have influenced asthma control and CPAP settings, which may be of clinical significance in future research aimed at optimizing symptom control in AOS patients. Moreover, due to the retrospective design of the study, there was no record of the time interval between asthma onset and the referral for sleep apnea investigation. Lastly, asthma control was assessed subjectively using the ACT questionnaire, without objective measures such as spirometry to evaluate the effects of OSA and CPAP in patients with AOS.

## 5. Conclusions

In conclusion, the findings of this study indicate not only the positive effect of CPAP therapy on patients with AOS but also that asthma control is negatively correlated with OSA severity and BMI, further supporting the bidirectional relationship between the two diseases. In addition, these results highlight the importance of screening for and treating sleep apnea in patients with asthma to improve sleep quality, optimize asthma symptoms control, and reduce the risk of future exacerbations. Nonetheless, further research on a larger scale is required to better understand these interactions, as well as the precise effect of CPAP therapy on patients with AOS.

## Figures and Tables

**Table 1:** Anthropometric characteristics of patients with asthma and OSA.

Anthropometric Characteristics	Patients with Asthma and OSA (Ν = 102)
Gender (males/females)	53/49
Age (years)	56.6 ± 12.8
Neck circumference (cm)	43.6 ± 4.6
Waist circumference (cm)	122.1 ± 15.5
Hip circumference (cm)	119.6 ± 20.3
WHR	0.90 ± 0.22
BMI (kg/m^2^)	38.2 ± 8
Smoking (Ν)	
Nonsmokers	45
Ex-smokers	29
Smokers	28
Menopause (Ν)	45

Abbreviations: BMI: body mass index; WHR: waist-to-hip ratio.

**Table 2:** Comparison of sleep characteristics of patients with uncontrolled, partially controlled, and well-controlled asthma.

	Patients with Uncontrolled Asthma (Ν = 27)	Patients with Partially Controlled Asthma (Ν = 58)	Patients with Well-Controlled Asthma (Ν = 17)	*p*
OSA severity (Ν/%)				
Mild	0	20 *	4	
Moderate	8	15	5	0.011
Severe	19	23 *	8	
TST (min)	308.6 ± 56.6	290.5 ± 62.3	287.2 ± 56.5	0.371
N1 (% TST)	23.8 ± 21.2	17.4 ± 14.1	20.4 ± 16.9	0.255
N2 (% TST)	56.5 ± 21.3	63.4 ± 18.2	59.8 ± 18.7	0.299
N3 (% TST)	8.7 ± 9.2	10.7 ± 8.8	9.6 ± 9.4	0.615
REM (% TST)	11 ± 8.6	8.5 ± 7.1	10.1 ± 6.8	0.322
Sleep efficiency (%)	77.5 ± 16.7	76.3 ± 14.7	76.7 ± 14.2	0.944
AHΙ (events/hour)	60.2 ± 31 * ^#^	27.8 ± 18.9	30.4 ± 18.8	<0.001
ODI (events/hour)	68.1 ± 34.3 * ^#^	32.9 ± 20.2	30.4 ± 17.9	<0.001
Aver SpO_2_ (%)	89.5 ± 4.1 *	92.1 ± 3.5	92 ± 1.7	0.007
Min SpO_2_ (%)	67.6 ± 13.4 * ^#^	76.3 ± 10	77.5 ± 8.3	0.001
T < 90% (%)	34.5 ± 28.9 *	15.3 ± 22.5	17.4 ± 25.1	0.005
Arousal index	25.3 ± 25.9	15.5 ± 18.2	25 ± 27.8	0.260
ESS score	12.4 ± 5 * ^#^	8.6 ± 4.9	7.5 ± 4.5	0.001

Abbreviations: AHI: apnea hypopnea index; Aver SpO_2_: average oxyhemoglobin saturation; ESS: Epworth sleepiness scale; Min SpO2: minimum oxyhemoglobin saturation; N1: sleep stage 1; N2: sleep stage 2; N3: sleep stage 3; ODI: oxygen desaturation index; REM: rapid eye movement; TST: total sleep time; T < 90%: time spent with oxyhemoglobin saturation <90%. *, *p* < 0.05 between patients with uncontrolled and partially controlled asthma; #, *p* < 0.05 between patients with uncontrolled and well-controlled asthma.

**Table 3:** Comparison of asthma control test scores and respiratory function between patients with uncontrolled, partially controlled, and well-controlled asthma.

	Patients with Uncontrolled Asthma (Ν = 27)	Patients with Partially Controlled Asthma (Ν = 58)	Patients with Well Controlled Asthma (Ν = 17)	*p*
FEV_1_ (% pred.)	89.9 ± 9.8	87.8 ± 20.2	87.3 ± 13.6	0.838
FVC (% pred.)	89 ± 14.9	88.3 ± 21.4	92.3 ± 17.2	0.750
FEV_1_/FVC (%)	81.6 ± 9.7	81.4 ± 9.4	80.2 ± 9.6	0.881
PEF (% pred.)	99.7 ± 23.8	87 ± 27.3	86.9 ± 26.5	0.102
ACT score	12.6 ± 2.1	17.4 ± 0.9	21.3 ± 1.7	<0.001

**Table 4:** Comparison of sleep characteristics between patients with and without asthma exacerbations.

	Patients Without Asthma Exacerbations (Ν = 58)	Patients with Asthma Exacerbations (Ν = 44)	*p*
OSA severity (Ν/%)			
Mild	17	7	
Moderate	17	11	0.159
Severe	24	26	
TST (min)	286.3 ± 66.2	305.8 ± 49.2	0.106
N1 (% TST)	19.3 ± 16.4	19.9 ± 17.3	0.859
N2 (% TST)	61.2 ± 18.9	60.7 ± 19.7	0.889
N3 (% TST)	10.4 ± 9.1	9.5 ± 8.8	0.618
REM (% TST)	9.1 ± 7.6	9.9 ± 7.5	0.566
Sleep efficiency (%)	74 ± 16.2	80.3 ± 12.6	0.038
AHΙ (events/hour)	31.6 ± 23.7	43.7 ± 28.7	0.022
ODI (events/hour)	34.6 ± 22.3	51.4 ± 33.8	0.006
Aver SpO_2_ (%)	92.2 ± 2.1	90.4 ± 4.8	0.021
Min SpO_2_ (%)	76.4 ± 9.8	71.3 ± 12.7	0.031
T < 90% (%)	15 ± 19.4	28.3 ± 31.1	0.016
Arousal index	17.7 ± 22	21.1 ± 21.7	0.555
ESS score	8.9 ± 4.9	10.1 ± 5.5	0.272

Abbreviations: AHI: apnea hypopnea index; Aver SpO_2_: average oxyhemoglobin saturation; ESS: Epworth sleepiness scale; Min SpO_2_: minimum oxyhemoglobin saturation; N1: sleep stage 1; N2: sleep stage 2; N3: sleep stage 3; ODI: oxygen desaturation index; REM: rapid eye movement; TST: total sleep time; T < 90%: time spent with oxyhemoglobin saturation <90%.

## Data Availability

Data available on request from the authors.

## Abbreviations

The following abbreviations are used in this manuscript:

OSA	Obstructive Sleep Apnea
ACT	Asthma Control test
CPAP	Continuous Positive Airway pressure
PSG	Polysomnography
AHI	Apnea–Hypopnea Index
GINA	Global Initiative of Asthma
AOS	Alternative Overlap syndrome
EDS	Excessive daytime Sleepiness
ESS	Epworth sleepiness scale
GERD	Gastroesophageal reflux
WMD	Weighted mean difference
CI	Confidence interval
